# Chronological Dynamics of Neuroinflammatory Responses in a High-Fat Diet Mouse Model

**DOI:** 10.3390/ijms252312834

**Published:** 2024-11-29

**Authors:** Heekyong R. Bae, Su-Kyung Shin, Ji-Yoon Lee, Seong-Su Choi, Eun-Young Kwon

**Affiliations:** 1 Department of Food Science and Nutrition, Kyungpook National University, Daegu 41566, Republic of Korea; baehee@knu.ac.kr (H.R.B.);; 2 Center for Food and Nutritional Genomics, Kyungpook National University, Daegu 41566, Republic of Korea; 3 Center for Beautiful Aging, Kyungpook National University, Daegu 41566, Republic of Korea

**Keywords:** neuroinflammation, obesity, systemic chronic inflammation, mitochondrial complex I activity, interferon-gamma

## Abstract

Obesity is known to affect various tissues and contribute to conditions such as neuroinflammation. However, the specific mechanisms and time-dependent progression of these effects across different tissues remain unclear. In this study, we monitored gene expression at intervals to examine the effects of a high-fat diet (HFD) on brain, liver, adipose, and muscle tissues in male C57/BJ mice, with a particular focus on neuroinflammation. Early inflammatory responses exhibit a progression that starts in the liver, extends to adipose tissue, and subsequently involves muscle and brain tissues. Although the brain did not show significant gene expression of inflammatory responses, mechanisms leading to neuroinflammation increased after 24 weeks, possibly through systemic chronic inflammation (SCI). Notably, mitochondrial complex I activity serves as a biomarker to indicate the inflammatory transition from the liver to adipose and other tissues caused by SCI. These similar gene expression dynamics were also observed in the hippocampus of Alzheimer’s patients and in an Alzheimer’s mouse model treated with a HFD. These results suggest that initially, the brain suppresses inflammatory responses, including interferon-gamma (IFN-γ), more than other tissues in response to a HFD. However, at the onset of SCI, the brain eventually exhibits inflammatory dynamics similar to those of other tissues. This underscores the significance of our findings, indicating that the early kinetics of chronic IFN-γ response and mitochondrial complex I activity inhibition serve as crucial biomarkers, emerging early in various conditions, including obesity and aging.

## 1. Introduction

The relationship between obesity and neuroinflammation is becoming increasingly clear through various studies. Obesity induces chronic low-grade inflammation, particularly in visceral fat, which releases inflammatory cytokines such as tumor necrosis factor alpha (TNF-α) and interleukin-6 (IL-6) [1]. These cytokines can cross the blood–brain barrier, potentially activating microglia, the brain’s innate immune cells, and triggering neuroinflammation [2]. This neuroinflammation is associated with neurodegenerative diseases such as Alzheimer’s, Parkinson’s, and depression [3]. Additionally, obesity-related insulin resistance affects brain insulin signaling, which may further exacerbate neuroinflammation and cognitive decline [4,5]. However, the exact mechanisms by which obesity induces neuroinflammation, as well as the precise causal relationship between obesity and neuroinflammation, remain unclear and require further research.

Mitochondrial dysfunction significantly influences neuroinflammation through several interconnected mechanisms [6,7], which include the production of excessive reactive oxygen species (ROS), dysregulated mitochondrial Ca^2+^ disturbance, and impaired mitophagy. Additionally, mitochondrial dysfunction results in the release of mitochondrial damage-associated molecular patterns (mtDAMPs), such as mitochondrial DNA (mtDNA) and cytochrome c, which activate immune receptors and stimulate the production of pro-inflammatory cytokines [8]. Mitochondrial dysfunction also leads to the activation of microglia, the brain’s resident immune cells, which release pro-inflammatory mediators, further exacerbating neuroinflammation [9].

Abnormal mitochondrial complex I activity is closely linked to neurodegenerative diseases such as Alzheimer’s disease (AD) [10]. Mitochondrial complex I is the first unit of the electron transport chain and plays a crucial role in ATP production [11]. When the activity of this complex is inhibited, ATP generation decreases, leading to insufficient energy supply to neurons. This energy deficit can cause neuronal dysfunction and death, which are key mechanisms in the pathology of AD. Moreover, under pathological conditions, inhibition of mitochondrial complex I leads to heightened ROS production, thereby increasing oxidative stress [12]. This oxidative stress is associated with the accumulation of amyloid-beta and abnormal phosphorylation of tau proteins, both of which are characteristic pathological changes in AD [13]. On the other hand, partial inhibition of mitochondrial complex I activity by complex I inhibitors has been proposed as a therapeutic strategy in AD [14], implying that excessive activation of complex I eventually leads to abnormalities in complex I activity.

Recent research has illuminated the critical role of mitochondrial translation in the context of AD [15,16]. It has been shown that RNA modifications, specifically, N1-methylation of adenosine in the NADH dehydrogenase subunit 5 (ND5) mRNA, disrupt mitochondrial protein synthesis, leading to mitochondrial complex I dysfunction in AD patients [17]. Furthermore, mitochondrial dysfunction often precedes the clinical symptoms of AD, indicating that early mitochondrial impairment could be a driving factor in the disease’s progression [16]. This includes altered expression of genes involved in mitochondrial biogenesis and energy metabolism, leading to defective mitochondrial translation and subsequent cellular energy deficits. Overall, these findings underscore the potential of targeting mitochondrial translation processes as a therapeutic strategy in AD, aiming to restore mitochondrial function and mitigate neuronal damage.

In this study, we aim to investigate the mechanisms by which obesity-induced mitochondrial dysfunction contributes to neuroinflammation and the development of neurodegenerative diseases. By examining the specific roles of mitochondrial complex I activity and mitochondrial translation processes, we seek to elucidate the pathways that link obesity, mitochondrial dysfunction, and neuroinflammation and to identify potential therapeutic targets to mitigate these effects.

## 2. Results

### 2.1. Tissue-Dependent Interferon-Gamma and Inflammatory Responses over Time in HFD-Fed Mice

**Figure 1:**
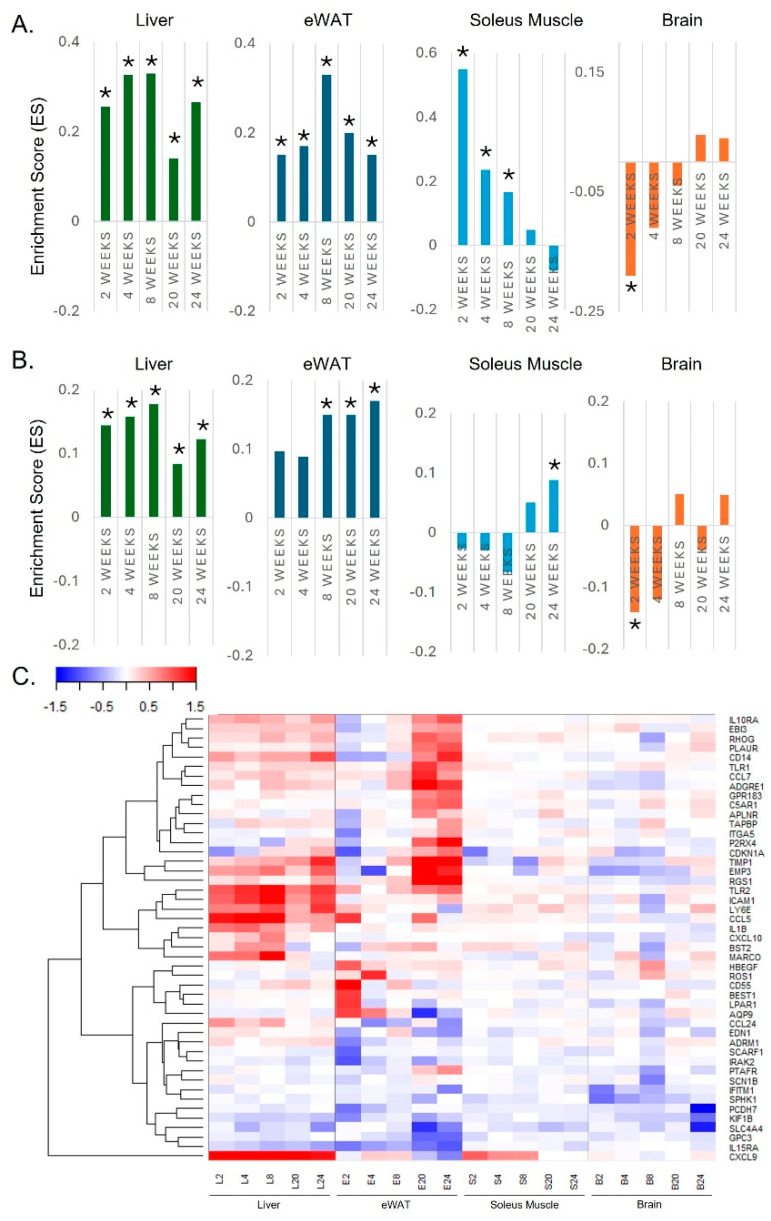
Tissue-dependent IFN-γ and overall inflammatory responses over time in HFD-fed mice, analyzed using pre-ranked GSEA. (**A**) Enriched IFN-γ responses over time in the liver, eWAT, soleus muscle, and brain of HFD-fed mice. (**B**) Enriched overall inflammatory responses over time in the liver, eWAT, soleus muscle, and brain of HFD-fed mice. (**C**) Heatmap of representative genes from a gene set related to inflammatory responses over time in the liver, eWAT, soleus muscle, and brain of HFD-fed mice. An asterisk denotes an FDR *q*-value < 0.05.

Our aim was to investigate how a high-fat diet affects different tissues over time, including the liver, epididymal white adipose tissue (eWAT), soleus muscle, and brain, based on IFN-γ and overall inflammatory responses, particularly given the importance of IFN-γ emphasized in our previous reports [18]. As shown in Figure 1A, a gene set related to IFN-γ responses was significantly down-regulated in the brain with the onset of a HFD, whereas this response increased in the soleus muscle from the beginning, similar to the liver and eWAT. In addition, we observed that a gene set related to inflammatory responses was sequentially activated in the liver, eWAT, soleus muscle, and brain, possibly due to SCI (Figure 1B). The specific genes present in each tissue are outlined in Figure 1C, which were selected from the top and bottom of the enriched gene sets using the same cut-off value. Taken together, our data provide new insights into the inflammatory transition from the liver to adipose tissue and from adipose tissue to SCI by observing tissue-dependent inflammatory responses over time. In particular, we observed that the brain shows a strong early protective response, which differs from other tissues.

### 2.2. Significant Mechanisms Altered by a HFD After 24 Weeks in the Brain

**Figure 2:**
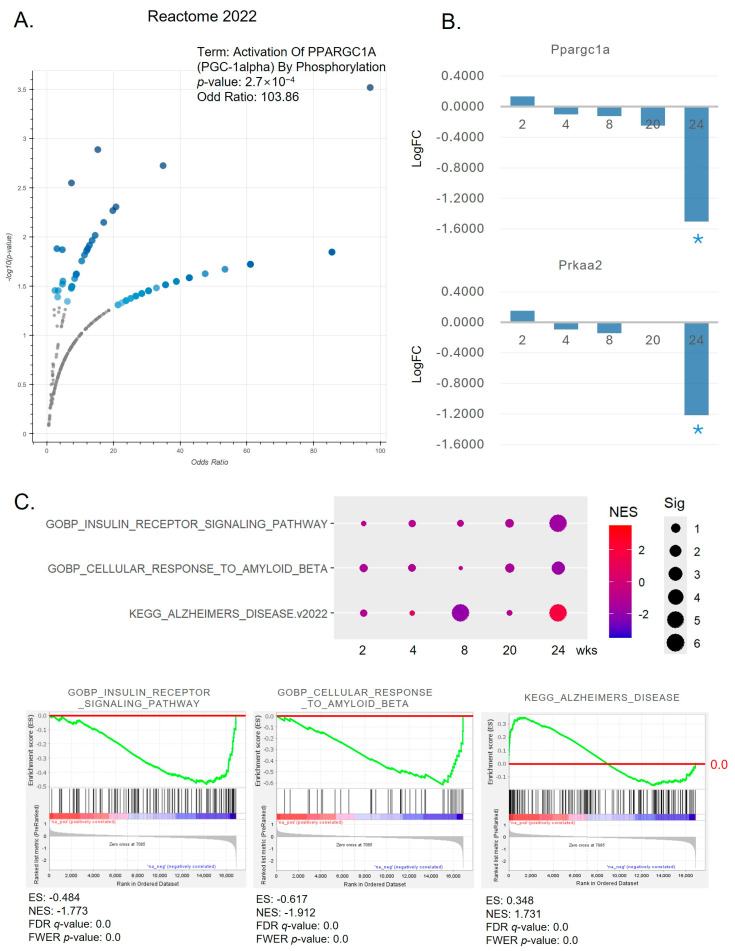
Disrupted pathways linked to neuroinflammation in the brain after 24 weeks of a HFD. (**A**) The volcano plot demonstrates the significance of the Reactome 2022 gene set, determined by −log(*p*-value), versus its odd ratio. Highlighted in larger blue points are terms deemed significant (*p*-value < 0.05), while smaller gray points indicate non-significant terms. The intensity of the blue color corresponds to the level of significance. (**B**) Gene expression profiles of Ppargc1a and Prkaa2 in the brain. The *y*-axis represents log-transformed fold changes. An asterisk denotes a *p*-value < 0.05. (**C**) A dot plot depicts the enriched gene sets associated with insulin receptor signaling, cellular response to amyloid-beta, and AD in the brain. The size of each dot signifies the significance level (Sig), shown as −log10(FDR *q*-value), while the color of the dot reflects the normalized enrichment score (NES). Smaller dots indicate non-significant pathways but still show trends. Each enrichment graph was created using GSEA. A red line marks the baseline (0.0) of the enrichment score.

In exploring the mechanisms of brain changes induced by a HFD after 24 weeks, distinct patterns were observed despite the lack of significant alterations in overall inflammatory responses. Regarding the brain-specific effects of a HFD, significant changes in mechanisms were detected starting from the 24-week mark. Figure 2A illustrates that among DEGs after 24 weeks, the Reactome 2022 database identified a gene set related to the activation of PGC1-α by phosphorylation as one of the most significant changes. Notably, the expression of Ppargc1a and Prkaa2 genes was significantly reduced after 24 weeks compared to other time points (Figure 2B). These findings were corroborated by pre-ranked GSEA based on changes in the entire gene set. As demonstrated in Figure 2C, gene sets associated with the insulin receptor signaling pathway, cellular response to amyloid-beta, and AD showed significant changes after 24 weeks. Specifically, the insulin receptor signaling pathway and cellular response to amyloid-beta gene sets were notably reduced, while the AD-related gene set was significantly increased.

### 2.3. Co-Regulation Between Mitochondrial Complex I Activity and Mitochondrial Translation Across All Tissues

**Figure 3:**
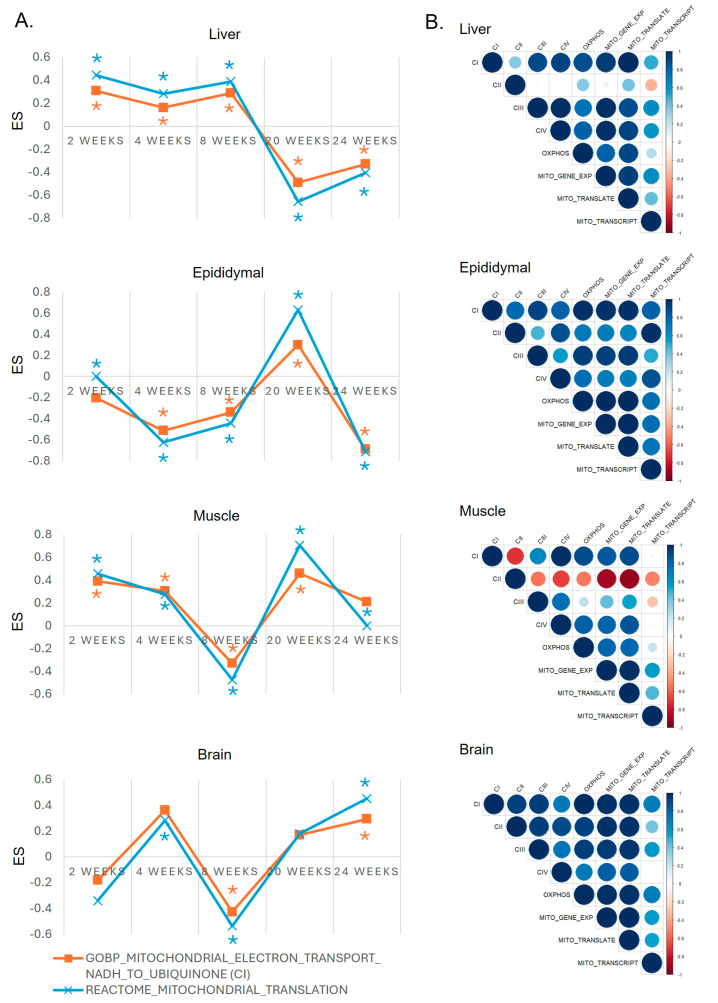
Co-regulation and correlation of mitochondrial processes and mitochondrial complex I activity in diverse tissues. (**A**) Co-regulation between mitochondrial translation gene sets and complex I activity in the soleus muscle and brain tissues. The *y*-axis indicates the enrichment score (ES), and an asterisk denotes an FDR *q*-value < 0.05. The blue line represents a gene set related to mitochondrial translation activity, while the orange line indicates a gene set associated with electron transport via Complex I. (**B**) Pearson correlation analysis among enriched gene sets of mitochondrial gene expression, translation, transcription, and mitochondrial complexes. The Pearson correlation coefficient and corresponding colors indicate the correlation strength and direction, with shades ranging from red for negative correlations to blue for positive correlations.

Similar to our previous findings in the liver and adipocytes, where gene sets associated with the mitochondrial translation process were co-regulated with mitochondrial complex I activity [19], this co-regulation was also observed in the soleus muscle and the brain, even though the two gene sets did not have overlapping genes (Figure 3A). Furthermore, correlation analysis between gene sets related to mitochondrial gene expression, mitochondrial translation, and mitochondrial transcription with mitochondrial complexes revealed that mitochondrial gene expression is primarily regulated during the translation process rather than the transcription process (Figure 3B). Therefore, our data suggest that the inhibition of mitochondrial complex I activity is specifically co-regulated with mitochondrial translation, thereby modulating mitochondrial function.

We previously demonstrated that the dysregulation of mitochondrial complex I activity in the liver coincides with increased inflammatory responses in eWAT [19], suggesting that similar kinetics might trigger SCI [19,20]. In this study, we aimed to investigate how these kinetics are related not only in the liver and adipocytes but also in muscle and brain tissues. As shown in Figure 3B, both soleus muscle and brain tissues exhibited a significant correlation between inhibited mitochondrial complex I activity and oxidative phosphorylation (OXPHOS) responses compared to other complexes. This suggests that chronic inflammation induced by IFN-γ, along with increased levels of other inflammatory factors such as TNF-α and IL-6, may lead to the inhibition of mitochondrial complex I activity.

### 2.4. Chronic Inflammation Induced by Obesity and Aging Linked to the Dysregulation of Mitochondrial Complex I Activity in AD

**Figure 4:**
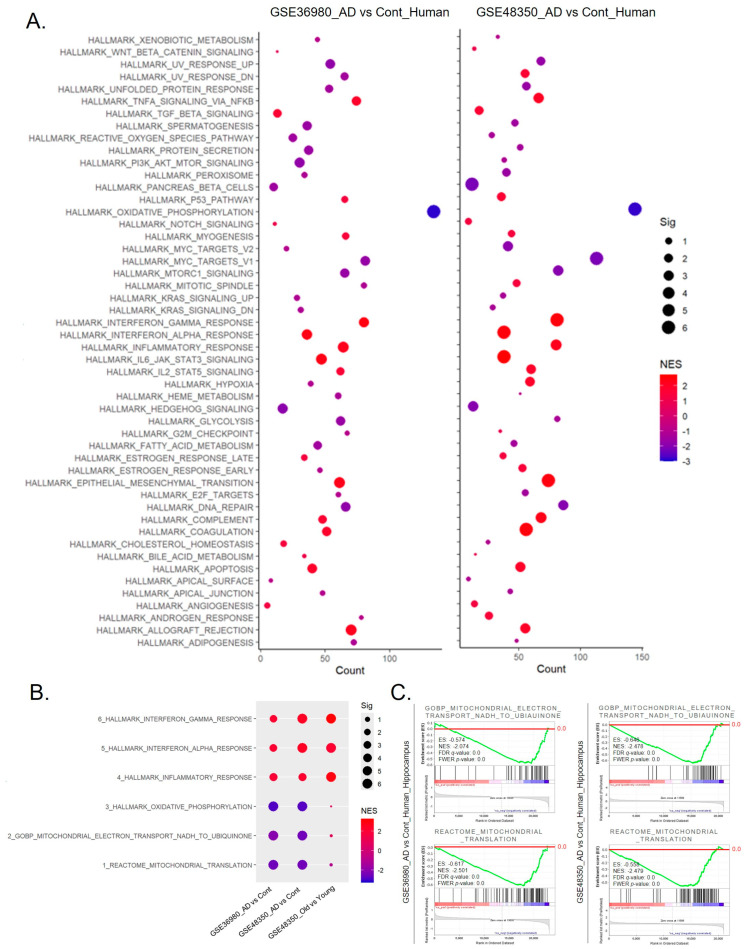
Comparative analysis of hallmark gene sets in the hippocampus of AD patients and age-matched controls. (**A**) Dot plots showing a 50 hallmark gene set analysis of hippocampus data from AD patients using genomic datasets GSE36980 and GSE48350. The size of each dot indicates the significance level (Sig), expressed as −log10(FDR *q*-value), while the color of the dot represents the normalized enrichment score (NES). (**B**) Dot plot showing enrichment of gene sets between older (>90) and younger (<40) healthy individuals, and between AD patients and age-matched controls (GSE36980, GSE48350). Smaller dots indicate non-significant pathways but still show trends. (**C**) Enriched graph patterns generated from GSEA, illustrating the suppression of mitochondrial complex I activity and mitochondrial translation processes in the hippocampus from AD patients. A red line marks the baseline (0.0) of the enrichment score.

We conducted a 50 hallmark gene set analysis on hippocampus data from AD patients using the genomic datasets GSE36980 and GSE48350. As shown in Figure 4A, the results were highly consistent between the two independently generated datasets. Specifically, gene sets related to IFN-γ, interferon-alpha (IFN-α), TNF-α, and IL-6 responses exhibited strong increases, while the OXPHOS response was markedly inhibited. In Figure 4B, when comparing responses between healthy individuals over 90 years old (old ages) and those under 40 (young ages), inflammatory responses showed a similar increase to that observed in AD patients. In contrast, mitochondrial electron transport to ubiquinone or mitochondrial complex I activity did not exhibit a significant decrease in AD patients, similar to the reductions seen in OXPHOS and the mitochondrial translation process. Figure 4C shows enriched graph patterns generated from GSEA, illustrating the suppression of mitochondrial complex I activity and mitochondrial translation processes in the brain. Additionally, in Supplementary Figure S1, the 50 hallmark gene set analysis revealed that the most significantly altered gene sets due to a HFD were those associated with IFN-γ and IFN-α in App (NL-F/NL-F) mice, a mouse model of AD.

## 3. Discussion

This study unveils significant insights into the chronological dynamics of neuroinflammatory responses induced by a HFD in a mouse model, providing a nuanced understanding of the tissue-specific progression of SCI and its implications for neuroinflammation and related pathologies. One of the key innovative findings of our study is the detailed mapping of the sequential activation of inflammatory responses across different tissues. We previously reported that inflammation initiates in the liver and sequentially affects eWAT [19]. In this study, we demonstrated how it sequentially impacts the muscle and, ultimately, the brain. This progression aligns with the concept of SCI, illustrating how chronic inflammation in one tissue can propagate to others, thereby amplifying the overall inflammatory burden. In particular, the pre-ranked GSEA method we employed revealed a noteworthy aspect: while traditional pathway analysis based on DEGs often exhibits high variation, our approach showed high consistency in interpreting changes induced by a HFD.

The early protective response observed in the brain, which eventually succumbs to inflammation after prolonged exposure to a HFD, indicates an adaptive mechanism that initially delays neuroinflammation but, ultimately, cannot prevent it. This pattern, potentially more pronounced than in other tissues, is likely due to the specialized role of microglia in neuroprotection compared to other tissue macrophages [21,22]. Furthermore, the rapid elevation of brain-derived neurotrophic factor (BDNF) responses in brain tissue suggests a brain-specific protective reaction [23]. Ramalho et al. reported that hypothalamic BDNF expression increases after 1 week on a HFD, normalizes by 2 weeks, and significantly decreases after 4 weeks of exposure to a HFD [24]. Similarly, our findings revealed that BDNF signaling, as characterized by the gene set of brain-derived neurotrophic factor (BDNF) signaling pathway from the WikiPathways database, was significantly down-regulated after 20 weeks of a HFD. Additionally, at the 24-week mark, when systemic inflammation became evident in eWAT, we observed a significant suppression of peroxisome proliferator-activated receptor gamma coactivator 1-alpha (PGC1-α) in the brain. This correlation highlights the need for further investigation into the interconnected mechanisms between systemic inflammation and neuroinflammation.

Microglia, the resident immune cells of the brain, can display pro-inflammatory (M1) and anti-inflammatory (M2) phenotypes, similar to macrophages in other tissues [25]. Recent studies indicate that microglia’s role in neurodegenerative diseases is not merely due to the shift in their polarization from M1 to M2. Comprehensive translatome analysis in brain immune cells using single-cell sequencing technology has identified the presence of specific pathogenic microglia populations in patients with AD [26,27]. This suggests that microglia do not simply oscillate between M1 and M2 states but exist as distinct subsets of cells with unique functions and characteristics [28]. Therefore, our results also imply that rather than a transition from M1 to M2 state, specific pathogenic populations of microglia may be generated in response to SCI.

Additionally, from a mechanistic perspective, our findings suggest that the shift of these cells from a protective to a pro-inflammatory state occurs at a specific transition point rather than gradually. To elaborate, inflammatory responses induced by a HFD initially manifest strongly in the liver, particularly affecting macrophages [19]. The inhibition of mitochondrial complex I activity in liver macrophages appears to mark the shift of inflammatory responses to eWAT. This eWAT-driven SCI is expected to exhibit similar kinetics and transition points in other tissues. This transition is also accompanied by a significant decrease in PGC1-α, underscoring its critical role at this juncture. Considering our observation that macrophage dysregulation was a key factor in the initial transition points in the liver and eWAT changes, it is plausible that similar kinetics apply to the brain, where microglial changes represent pivotal transition points. Moving forward, it is essential to investigate how different microglia subpopulations change in association with a HFD, using techniques such as single-cell sequencing.

Our findings identify dysregulated mitochondrial complex I activity as a critical biomarker for the inflammatory transition across tissues induced by a HFD. Furthermore, our data demonstrate that mitochondrial complex I activity and mitochondrial translation processes are co-regulated across all tissues, suggesting that impaired complex I activity directly disrupts mitochondrial translation, or vice versa, thereby driving pathogenic processes. Notably, seven essential components of complex I, specifically, ND1, ND2, ND3, ND4, ND4L, ND5, and ND6, are encoded by the mitochondrial genome (mtDNA) and synthesized within the mitochondrial matrix [29]. These mtDNA-encoded subunits play critical roles in the assembly and function of complex I. In this context, recent reports indicate that abnormalities in mitochondrial translation are linked to mitochondrial respiratory chain dysfunction, potentially contributing to the onset of various diseases [30,31]. These insights provide a strong rationale for the development of therapeutic strategies focused on boosting mitochondrial complex I activity and optimizing mitochondrial translation.

A remarkable aspect of our study is the parallel drawn between the inflammatory and mitochondrial dysregulation observed in HFD-fed mice and AD patients. Significant increases in IFN-γ, IFN-α, TNF-α, and IL-6 responses, along with marked inhibition of OXPHOS responses, support the hypothesis that systemic chronic inflammation, exacerbated by metabolic conditions such as obesity, can contribute to the development and progression of neurodegenerative diseases. Notably, in the absence of disease, aged individuals exhibited significant inflammatory responses without significant inhibition of mitochondrial complex I activity. This suggests that low-grade inflammation due to aging in the brain could eventually lead to the inhibition of complex I activity. Therefore, both low-grade inflammation caused by aging and obesity may follow similar kinetics in contributing to the development of AD.

A critical question in understanding the pathogenesis of obesity-related diseases is how SCI caused by obesity triggers neuroinflammation in the brain. Our study highlights the pivotal role of IFN-γ as a key mediator in this process. As previously mentioned, our group’s prior findings indicated that low but chronic IFN-γ expression induced by a high-fat diet (HFD) is linked to dysregulated mitochondrial complex I activity in the liver. This mitochondrial dysfunction appears to trigger a cascade of inflammatory signals, spreading inflammation from white adipose tissue (WAT) into systemic circulation. Chronic exposure to IFN-γ has been shown to critically disrupt mitochondrial complex I activity, as supported by studies in myocytes, where IFN-γ exposure led to fatigue-like symptoms [32]. This suggests that IFN-γ-mediated disruption of mitochondrial complex I activity may serve as a general trigger for the pathogenesis of various immune-related disorders.

Chronic IFN-γ, possibly originating from WAT, may serve as a critical trigger for inflammatory responses in distant tissues, including the brain. Similarly, our data demonstrate that in App (NL-F/NL-F) mice, a model of AD, a HFD induced an IFN-γ response, identified as the top pathway (Supplementary Figure S1). This underscores the potential of IFN-γ as a key link between obesity-induced SCI and neuroinflammation. The systemic impact of IFN-γ may stem from low but chronic levels of IFN-γ in circulation, which can activate inflammatory pathways in the brain. Additionally, an alternative mechanism involves the role of natural killer (NK) cells, which are a major source of IFN-γ. NK cells have been reported to become activated in WAT under conditions of obesity-induced SCI and can migrate through the bloodstream [33]. Upon reaching distant tissues, including the brain, these activated NK cells may further amplify inflammation by secreting IFN-γ.

One important aspect to discuss is the dual role of IFN-γ in regulating mitochondrial complex I activity, particularly in Th1 cells and macrophages, and its implications for AD pathology. IFN-γ plays a context-dependent role in mitochondrial function. Acutely, it enhances complex I activity by promoting mitochondrial biogenesis and improving electron transport chain (ETC) efficiency, supporting Th1 differentiation and cytokine production [34]. In macrophages, increased complex I activity fuels M1 polarization, supplying ATP and ROS necessary for initiating inflammatory signaling pathways [35]. However, prolonged exposure to IFN-γ under chronic or pathological conditions can disrupt mitochondrial function, leading to complex I inhibition, excessive ROS production, oxidative stress, and the release of mitochondrial damage-associated molecular patterns (mtDAMPs) [36]. These changes amplify inflammation and tissue damage, contributing to chronic neuroinflammation seen in AD.

Our findings align with existing evidence that mitochondrial complex I dysfunction is a critical driver of neurodegeneration and cognitive decline in AD [10,37]. Well-known complex I inhibitors, such as rotenone, are often used to model Parkinson’s disease and have also been studied in the context of early-stage AD [38,39]. Interestingly, there is controversy surrounding the role of complex I inhibition in AD. Partial inhibition of complex I has been shown to alleviate AD symptoms by reducing excessive ROS production while preserving ATP generation capacity [40]. To explain these conflicting roles of complex I activity in AD, we applied the previously described dual role of IFN-γ in modulating complex I activity. During acute phases, IFN-γ boosts complex I activity by stimulating mitochondrial biogenesis and enhancing the efficiency of the ETC, thereby facilitating Th1 cell differentiation. However, prolonged pro-inflammatory responses can lead to metabolic switching from OXPHOS to glycolysis for rapid ATP production [41]. In this process, sustained inhibition of complex I may result in persistent suppression of mitochondrial translation, ultimately causing complex I dysregulation. Thus, while partial inhibition of complex I initially mitigates AD pathology, possibly by dampening early pro-inflammatory responses, complex I dysregulation plays a significant role in driving AD pathogenesis over time.

Finally, the identification of early biomarkers, such as chronic IFN-γ response and mitochondrial complex I activity inhibition, offers valuable insights for developing therapeutic strategies. Targeting these pathways could potentially delay or prevent the onset of neuroinflammation and its associated cognitive decline. Our findings emphasize the need for therapeutic approaches that address both systemic and localized inflammation, particularly focusing on mitochondrial function modulation. Future research should aim to elucidate the precise mechanisms driving the transition from systemic to neuroinflammation. Longitudinal studies involving various stages of exposure to a HFD and aging will help clarify the temporal dynamics and causal relationships. Additionally, exploring the therapeutic potential of modulating mitochondrial function and inflammatory pathways in both obesity and neurodegenerative disease models could pave the way for novel treatments. The dysregulation of complex I activity, rather than its simple inhibition, is a critical indicator of SCI progression. Therefore, efforts should focus on developing methods to detect and utilize this dysregulation early in clinical practice considering its potential as a key marker for intervention.

## 4. Materials and Methods

### 4.1. Data Acquisition and Processing

To study the effects of a HFD on various tissues, including the liver, white adipose tissue, soleus muscle, and brain, we conducted a time-course analysis using male C57BL/6J mice obtained from the Jackson Laboratory at 4 weeks of age. The mice, with a minimum of *n* = 3 per group, were fed a semi-purified diet containing 39.2% kcal from fat. The following time points were selected for analysis across all tissues: 0, 2, 4, 8, 20, and 24 weeks. Gene expression microarray datasets were generated using Illumina MouseWG-6 v2.0 Expression BeadChip microarrays (Illumina, San Diego, CA, USA). The liver and white adipose tissue data were stored under the accession number GSE39549 in the Gene Expression Omnibus (GEO). To identify differentially expressed genes (DEGs) between the HFD and normal diet (ND) groups at each time point, we used LIMMAs (Linear Models for Microarray Data) for differential expression analysis. 

To investigate the mechanistic similarities between obesity, aging, and the development of AD, we utilized data from two independent studies available in the GEO database: GSE36980 and GSE48350. Briefly, GSE36980 aimed to identify molecular changes in AD brains through interspecies comparative microarray analysis. RNA samples from postmortem human brain tissues donated for the Hisayama study were used, covering the gray matter of the frontal cortex, temporal cortex, and hippocampus. High-quality RNA samples underwent microarray analysis using the Affymetrix Human Gene 1.0 ST platform (Affymetrix, Santa Clara, CA, USA). This study included a total of 80 postmortem brain samples, focusing on AD and AD-like disorders. Gene expression profiles were analyzed across these brain regions, revealing significant alterations, particularly in the hippocampi of AD brains [42]. In GSE48350, microarray data were collected from normal controls (aged 20–99 years) and AD cases across four brain regions: hippocampus, entorhinal cortex, superior frontal cortex, and post-central gyrus. AD cases in this dataset were processed concurrently with control cases (young and aged) included in GSE11882, which contains data exclusively from normal control brains. In our comparative analysis, we specifically focused on hippocampus data extracted from both studies. By narrowing our scope to this brain region, we aimed to elucidate consistent molecular signatures and potential mechanistic links associated with AD pathology across different experimental contexts. This approach allowed us to explore the intersection of obesity, aging, and AD through comprehensive gene expression profiling in the hippocampus, thereby providing deeper insights into the shared molecular pathways underlying these complex relationships.

### 4.2. Pre-Ranked GSEA

Pre-ranked Gene Set Enrichment Analysis (GSEA) determineswhether specific gene sets are significantly enriched in a ranked gene list, indicating their association with a particular biological condition or pathological phenotype.. This approach was pivotal in our study, where we applied it to gene expression datasets from liver and adipose tissue, specifically focusing on fold change values across different time points. The algorithm calculates enrichment scores (ESs) and normalized enrichment scores (NESs) using permutation testing (1000 permutations), where the significance is assessed based on stringent criteria (*p*-adjust < 0.05, FDR *q*-value < 0.05 or 0.001). To identify enriched biological pathways associated with our phenotype, we utilized annotated gene collections from MSigDB v7.4, particularly leveraging hallmark gene sets known for their established roles in defining biological functions and pathways. Furthermore, our study involved a comprehensive curation of gene sets from various authoritative public databases such as Mouse Genome Informatics (MGI), Kyoto Encyclopedia of Genes and Genomes (KEGG), and Reactome. These curated gene sets enabled us to perform detailed analyses, validating our findings across multiple datasets and gene sets independently. This rigorous validation process ensured the reliability and robustness of our results, providing deeper insights into the molecular mechanisms underlying the observed gene expression changes in liver and adipose tissue.

### 4.3. Statistical Analysis and Visualization

For our statistical analysis and visualization, we employed the Analysis ToolPak in Excel to create graphs and explore our data. This Excel add-in offers essential statistical functions and visualization tools that helped us summarize our dataset effectively. To assess the significance of our findings, we conducted multiple *t*-tests and ANOVA using the Excel’s Analysis ToolPak add-in for Microsoft Office 365, 2023. The *t*-tests were used to compare means between two groups, while ANOVA allowed us to compare means across multiple groups, helping us pinpoint significant differences in our data. We established statistical significance at the conventional threshold of *p* < 0.05, ensuring the reliability of our conclusions. For more sophisticated data visualization, we turned to R (version 4.3.2), a powerful statistical computing language. Specifically, we utilized R packages like ggplot2 to create detailed dot plots. These visualizations offered clear insights into our data, highlighting important patterns and distinctions that numerical analysis alone might not reveal.

## Data Availability

The data used in this study are publicly available and can be accessed under the accession numbers GSE39549, GSE36980, and GSE48350. The authors declare that all relevant data supporting the findings of this study are available in the paper and its Supplementary Information files or can be obtained from the corresponding authors upon request. The brain and soleus muscle data also can be obtained from the corresponding authors upon request

## Supplementary Materials

The following supporting information can be downloaded at: https://www.mdpi.com/article/10.3390/ijms252312834/s1.
