# Supplementary material for: Chronological Dynamics of Neuroinflammatory Responses in a High-Fat Diet Mouse Model
Source: Int J Mol Sci. 2024 Nov 29;25(23):12834. doi: 10.3390/ijms252312834 (PMC11640818; doi:10.3390/ijms252312834)
Supplement: Supplementary file 1 [file ijms-25-12834-s001.zip › ijms-3314093-supplementary.pdf]

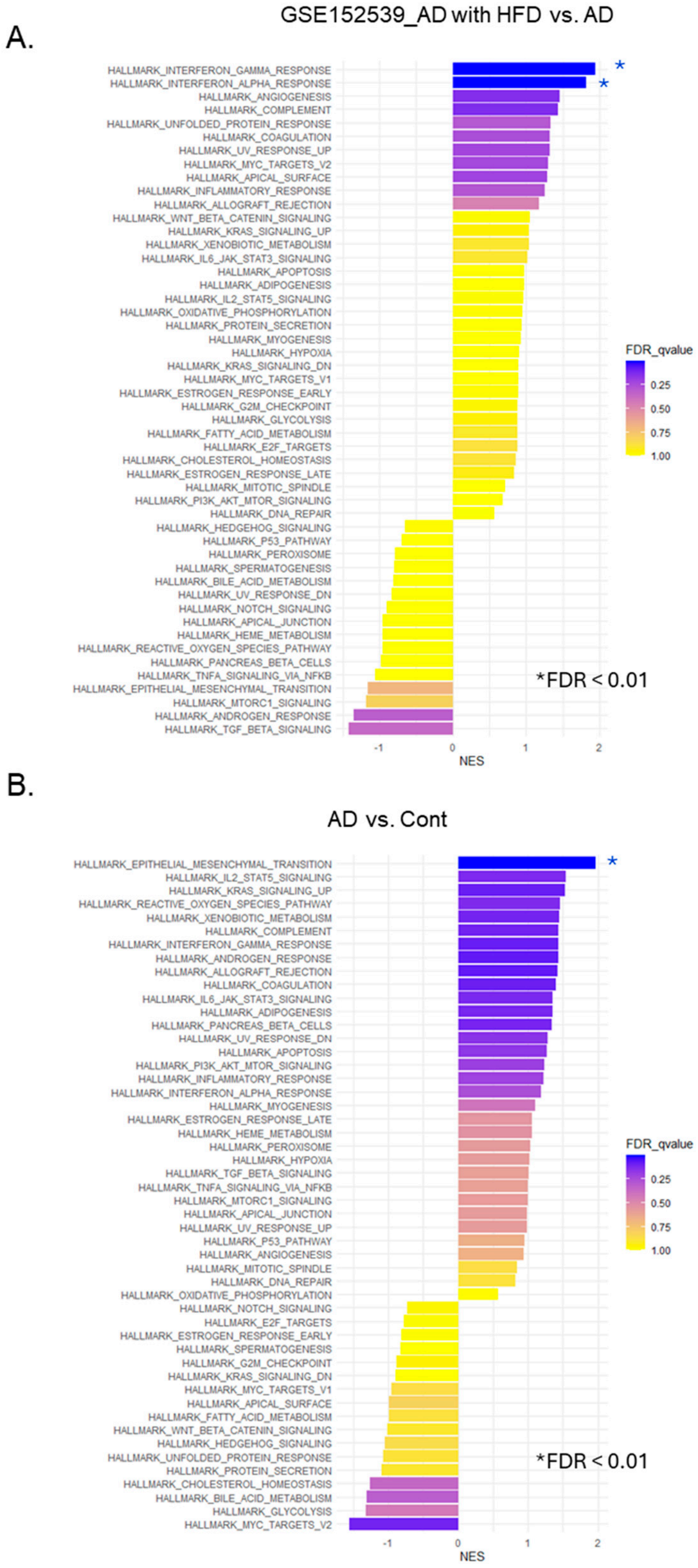

Supplementary Figure S1. Enrichment analysis of HFD effects on (NL-F/NL-F) mice, an AD model, using hippocampal microarray data from the GSE152539 dataset. 50 hallmark gene set analysis comparing HFD-fed mice to normal diet-fed App (NL-F/NL-F) mice (A). 50 hallmark gene set analysis comparing App (NL-F/NL-F) mice to control littermates (B).
